# Are there kinematic and kinetic parameters correlated with racket velocity during the tennis serve? A preliminary comparison between a slow and a fast serve for performance improvement

**DOI:** 10.3389/fspor.2024.1451174

**Published:** 2024-10-09

**Authors:** Philippe Gorce, Julien Jacquier-Bret

**Affiliations:** ^1^ International Institute of Biomechanics and Occupational Ergonomics, Hyères, France; ^2^ Université de Toulon, Toulon, France

**Keywords:** tennis serve, trophy position, racket low point, ball impact, cocking phase, acceleration phase, coaching, performance

## Abstract

**Introduction:**

The tennis serve is a complex motion with numerous rotations which are important to manage for performance. The main aim of this study was to investigate kinematic parameters, including the evolution of the center of gravity, and kinetic parameters correlated with racket velocity over all phases of the tennis serve. The secondary objective was to find out which of the correlated parameters differed between a slow and a fast serve. The advantage of such an approach would be to propose biomechanical parameters that coaches and teachers could use to optimize performance or learn how to serve.

**Methods:**

Quantitative analysis was carried out on 5 flat serves performed by four ranked players using an optoelectronic system (82 markers located on whole body and racket) composed of 10 infrared cameras (150 Hz) and two force platforms (750 Hz).

**Results:**

A descriptive statistical analysis highlighted 11 very large and almost perfect correlations with racket velocity: vertical ground reaction force of back foot in release backward, trunk axial rotation during loading phase, back and front knee flexions, dominant shoulder and hip mediolateral rotation during cocking phase, and center of gravity vertical velocity, dominant shoulder medial rotation velocity, dominant elbow flexion, trunk flexion/extension and axial rotation during acceleration phase. Differences were observed for some of the correlated parameters between slow and fast serve.

**Discussion:**

Consequently, all these correlated kinematic and kinetics parameters constitute information that coaches, instructors and athletes can use to improve, optimize or teach the tennis serve.

## 1 Introduction

In tennis, mastery of the serve is essential to performance. It is crucial to control the trajectory and velocity of the ball in order to surprise the opponent and gain an advantage from the opening serve. Knowing the kinematics that characterize the movement is therefore a key factor in improving technique and performance. Studies first investigated the effect of several parameters that could influence the kinematics of the serve such as the side of the serve (ad vs. deuce), the choice of trajectory (center line or outer part of the service area), the type of serve, or even the stance style. For example, Fett et al. showed a greater shoulder internal rotation velocity on the deuce side (2,028 ± 332°/s) than on the ad. side (1,970 ± 276°/s) (1). Reid et al. showed a higher knee extension velocity in the foot-up technique (9.3 ± 1.2 rad/s) than in the foot-back stance (7.2 ± 0.9 rad/s) (2). Reid et al. also showed a higher racket velocity during a flat serve (43.2 ± 3.1 m/s) compared to a kick serve (40.3 ± 2.9 m/s) (3).

More specifically, the tennis serve has been decomposed into stages and phases. Kovacs and Ellenbecker (4) described the serve through three phases with eight stages: preparation phase (with start, release, loading, and cocking stages), acceleration phase (acceleration and contact stages) and follow-through phase (with deceleration and finish stages). The various stages are characterized by five key points identified through certain kinematic parameters: (1) initial position with the racket at rest; (2) ball release when the ball leaves the non-serving hand; (3) trophy position with minimum vertical elbow position and maximum knee flexion; (4) racket low point when lateral shoulder rotation is maximum and the racket head points downwards; (5) ball impact.

Several kinematic studies have been carried out on the tennis serve at these key points. Fett et al. (1) studied the position of the trunk and feet in relation to the service line during ball release for each side of the serve. Some other studies have focused on trophy position and investigated peak knee flexion reported between 47 ± 21° and 81 ± 8° as a function of the type of serve [flat, slice, and top spin (5),], its finishing form [normal vs. arabesque (6)] or the age of the players [children, teenagers, and adults (7)]. Other authors have studied the evolution of lateral flexion shoulder-pelvis separation angle as a function of serve type [flat: 31.5 ± 7.3° vs. kick: 31.6 ± 7.5° (3),], stance style [foot up: 30.5 ± 6.4° vs. foot back: 31.1 ± 7.0° vs. low lower limb involvement: 32.1 ± 4.1° (2),] or during a successful or faulty serve in three groups of subjects [23 ± 2° to −31 ± 7° (8),]. The upper limb was also the subject of several studies. Tubez et al. (7) worked with a group of children, teenagers and adults to assess shoulder abduction (67 ± 24° to 88 ± 16°), elbow flexion (85 ± 17° to 107 ± 30°) and wrist flexion (2 ± 10° to 16 ± 11°) during trophy position (7). At racket low point, maximum shoulder external rotation has been the most reported parameter in the literature, with values ranging from 115 to 170° (9–12). Abrams et al. (13) and Reid et al. (3) studied the effect of serve type on flexion (flat: 8.3 ± 5.5°; kick: 16.3 ± 4.9°; slice: 14.7 ± 5.0°) and inclination (flat: 31.5 ± 7.3°; kick: 31.6 ± 7.5°) of the trunk. Fett et al. (1) proposed an in-depth analysis of racket low point by studying the effect of serve side on trunk angles, i.e., flexion (ad: 44.0 ± 10.6°; deuce: 44.2 ± 1.3°), and inclination (ad: 19.2 ± 6.5°; deuce: 19.4 ± 5.8°), and elbow flexion (ad: 132.2 ± 10.4°; deuce: 132.7 ± 9.8°). The time of impact with the ball is well documented in the literature. Elbow flexion (10°–45°) and shoulder elevation (92°–150°) have been extensively studied under a wide range of conditions (age, gender, stance style, effect of service type) (8, 14, 15). Shafizadeh et al. (16) reported values for the neck (flexion: 5°–8°; inclination: 20°–22°; axial rotation: 20 to 30°) and trunk (flexion: 29° and 33°; inclination: 8°–10°; axial rotation: 5 to 7) with and without an opponent. Values for the lower limbs, i.e., hip (29°–33°), knee (6°–20°), and ankle (30°–50°) have also been reported as a function of sex or age (11, 17).

Since the serve is a dynamic sequence whose chain begins with knee and hip extension (18), some authors have studied the distribution of ground reaction forces during the serve using force platforms. Elliott and Wood (19) studied the effect of stance style (foot-up and foot-back) on vertical and horizontal ground forces. The authors created a temporal profile during service and compared the two techniques for 3 instants. Bahamonde and Knudson (20) studied the same ground forces for both stances, adding two types of serve. No difference was observed between the two types of serve. On the other hand, foot-up stance allowed players to generate greater vertical force than foot-back technique (2.1 vs. 1.5 body weight), enabling them to generate a greater moment of rotation and therefore greater racket velocity. All these studies have compared kinematic or kinetic parameters, usually at key moments, but without necessarily making a direct link with the total temporal sequence of the gesture and therefore with performance.

Other studies have linked analysis to serve performance through ball or racket velocity. Several authors have carried out kinematic studies as it is the biomechanical variables that characterize the serve technique. Studies have also investigated the contribution of different joint regions. Tanabe and Ito (21) showed that the largest proportion of racket velocity was generated by the shoulder (41%), followed by the wrist (32%). Bahamonde (22) showed that the contribution of the trunk's axial rotation (around the vertical axis) is greatest in the acceleration phase, during which the racket acquires its maximum velocity before ball impact (4).

Hornestam et al. (23) compared racket velocity between two groups with different knee flexion. The authors showed that too little knee flexion (55.6 ± 8.6° vs. 74.7 ± 5.9°) resulted in a 3.3 km/h reduction in racket velocity. More recently, a study was carried out on the correlations between kinematic parameters and racquet velocity during cocking and acceleration (24). The knowledge provided by these studies enables the formulation of recommendations for training to optimize the serve performance in tennis. However, these analyses did not consider the joint organization required to perform the entire serve movement.

Given the complexity of the gesture (numerous rotations and wide ranges in all three planes), few studies have considered the entire serve motion. Three studies have equipped players to study the full body kinematics. Reid et al. (3) used 62 markers to analyze 8 joint angles and 3 velocities at a single key moment in the serve. Fett et al. (1) used 86 markers to propose 12 angles, 1 distance and 7 angular velocities peak at Start, during preparation, propulsion and at ball impact. Jacquier-Bret and Gorce (24) used 82 markers to measure 28 joint angles at BI, as well as the temporal evolution of 13 angular parameters correlated with racquet velocity or acceleration during cocking and acceleration stages. In-depth knowledge of all kinematic variables during the serve is essential to understand the whole motion and identify parameters that can be used by coaches to improve serve performance.

The primary aim of this study was to find out whether there were any parameters correlated with racket velocity across all phases of the serve. The secondary objective was to find out which of the correlated parameters differed between a slow and a fast serve. The advantage of such an approach would be to propose biomechanical parameters that coaches and teachers could use to optimize performance or learn how to serve.

## 2 Materials and methods

A sample of four players performed a series of flat tennis serves in the laboratory. Full-body kinematics was captured with an optoelectronic system. Ground reaction forces were obtained using force platforms. From these data, center of gravity displacements and velocities, ground reaction forces and joint angles and velocities were computed. The levels of correlation between these parameters and racket velocity during the different phases of the serve were investigated. Parameters with high correlations were used to compare slow and fast serves from the same player.

### 2.1 Participants

Table 1 provides demographic data on the sample studied. All subjects volunteered to take part in the experiment, and none suffered from any pathology or injury that might impair serve performance. All of them were right-handed, belonged to the same club, had the same coaches and played an average of 15 h of tennis a week. The complete protocol and objectives were presented before the beginning of the study, and each player gave written informed consent before participating. The protocol was approved by the Ethics Committee of the International Institute of Biomechanics and Occupational Ergonomics (IIBOE23-E74) and was in agreement with the Helsinki Declaration (25).

**Table 1:** Demographic data of the tennis players.

	Female	Male	All
Participants	*n* = 2	*n* = 2	*n* = 4
Age	16.5 ± 0.7	19.0 ± 2.8	17.8 ± 2.2
Height	1.59 ± 0.03 m	1.72 ± 0.02	1.66 ± 0.08
Weight	54.0 ± 5.6	59.0 ± 2.8	56.5 ± 4.6
BMI	21.0 ± 1.5	19.6 ± 0.8	20.3 ± 1.3
Rank	First series in French national ranking		

### 2.2 Experimental design

The player faced a wall located 11.88 m in front of him/her, onto which a net topped by a target area was projected to reproduce the conditions of a tennis court. The task was to perform a series of flat serves on the advantage side. The player was asked to serve only “first balls” at maximum velocity. Each player used his own racket and foot stance.

First, the subject carried out a 15-min warm-up session with his own racket to avoid injury and to familiarize him/herself with the measurement environment. Following warm-up, the tennis player was fitted with 56 anatomical markers (diameter: 14 mm) positioned on specific anatomical points of the whole body identified by palpation following the recommendations of the International Society of Biomechanics (26, 27). Due to the complexity of the movement (large amplitude, high velocity and numerous rotations in space), 18 technical markers were added on the arms, forearms and thighs in order to generate information redundancy and thus be able, if necessary, to interpolate the trajectories of anatomical markers obscured during service. To analyze racket kinematics, 8 markers were placed around the sieve and on the handle without interfering with the grip. Once equipped, the subject returned to face the wall to perform the series of flat serves. Each serve was followed by 1 min's rest. The player continued to serve until 5 trials had reached the target and were exploitable, i.e., with the minimum number of occultations.

Ten M5 infrared optoelectronic cameras (Qualisys AB, Gothenburg, Sweden, acquisition frequency at 150 Hz, resolution: 2,048 × 2,048 with 1 MPixel, 3D resolution: 0.07 mm) were used to record the trajectories of the 82 markers. Reaction forces along the anteroposterior, mediolateral and vertical axes were recorded throughout the serve using two force platforms (600 × 400 mm Kistler Type 9260AA with 5695A DAQ, Switzerland, 750 Hz, sensitivity: 2 mV/N, linearity: 0.5% of full scale output, maximum center of pressure error <2 mm, relative standard deviation of repeatability: 0.03% of full scale output). The subject was asked to start with one foot on each platform and then to execute the serve with his or her foot technique, i.e., foot-up or foot-back. The 10 cameras and the two platforms were connected to an electronic device that synchronized the recordings and data. Qualisys Track Manager software (v2020.3 build 6,020—Qualisys AB, Gothenburg, Sweden) (28, 29) was used to analyze reaction forces in three planes, 3D body tracking, automatic marker labeling, interpolation procedures, and anatomical markers reconstruction in case of loss. Finally, a digital camera positioned in the sagittal plane filmed all serves to complete the identification of key points of the tennis serve and to analyze ball position. Before each session, a calibration procedure was carried out according to the manufacturer's recommendations, using its software and hardware.

### 2.3 Data processing

For each trial recorded during the protocol, a processing phase was carried out to ensure the presence of markers throughout the entire movement. When a short occultation was detected, a cubic spline function from Qualisys Traker Manager was used to reconstruct the trajectory. For longer occultations, a geometric reconstruction using the technical markers was performed. Once the trajectories were fully available, the data were exported to Matlab (R2023a Update 5, v9.14.0.2237262, The Mathworks, Natick, MA, USA).

First, a Butterworth anti-aliasing low-pass filter (order 2, with a cut-off frequency of 8 Hz) was applied. Then, an anatomical marker constructed from the 3D coordinates of the markers was attached to each of the 15 body segments considered: head, trunk, pelvis, right and left arm, forearm and hand, right and left thigh, leg and foot, in agreement of ISB recommendations. Each rotation matrix between two consecutive segments was computed at each instant of movement, and joint angles were extracted from the rotation matrices using ISB rotation sequences. The YXY shoulder rotation sequence recommended in the ISB has been replaced by the XZY sequence (X: anteroposterior axis pointing forward, Y: vertical axis pointing upward, and Z: mediolateral axis pointing to the right) proposed by Bonnefoy-Mazure et al. (30). The authors showed that this sequence was more suitable for studying shoulder movements during the tennis serve.

A total of 33 degrees of freedom were considered in the analysis. Nine were associated with the axial skeleton: neck and trunk flexion (−)/extension (+), pelvis anteversion (−)/retroversion (+); neck, trunk, and pelvis left (−)/right (+) inclination; neck, trunk, and pelvis left (+)/right (−) rotation. The upper limbs counted 7 joint angles each: shoulder flexion (+)/extension (−), shoulder abduction (−)/adduction (+), shoulder medial (+)/lateral (−) rotation, elbow flexion (+), forearm pronation (+)/supination (−), wrist flexion (+)/extension (−), and wrist radio (−)/ulnar (+) deviation. The lower limbs contained 5 joint angles each: hip flexion (+)/extension (−), hip abduction (−)/adduction (+), hip medial (+)/lateral (−) rotation, knee flexion (−), and ankle flexion (+)/extension (−). The angular velocity and angular acceleration of each joint were computed for each trial.

Based on 3D marker coordinates and anthropometric tables (31), the 3D position, velocity and acceleration of the player's center of gravity (CG) was computed at each instant of the serve. Based on the work of Kovacs et al. 2009 (4), six key points were defined for analyzing the tennis serve: (1) The player's initial position (Start), i.e., racket pointing forward and ball in contact with the racket at rest; (2) Backward position (BP), which corresponds to the player's posture when the CG is furthest back on the anteroposterior axis; (3) Ball release (BR), which corresponds to the instant when the ball is released by the non-serving hand; (4) Trophy position (TP) corresponding to the moment of maximum knee flexion; (5) Racket low point (RLP) corresponding to the moment when the racket tip head is at its lowest altitude behind the back (pointing toward the ground); (6) Ball impact (BI) defined by the instant of contact between the ball and the racket. BI was obtained from video data after manual synchronization with the optoelectronic system, since no marker was placed on the ball. These key points define four phases of the serve: release, in two parts (backwards from Start to BP, and forwards from BP to BR), loading (from BR to TP), cocking (from TP to RLP), and acceleration (from RLP to BI) (4).

### 2.4 Statistical analysis

A descriptive analysis including a correlation analysis was conducted. Linear correlations between racket speed and all other parameters (3D position, velocity and acceleration of the CG and 33 joint angles as well as the reaction forces of both feet along the three axes) were studied for each of the 5 phases of the tennis serve using Statistica software (version 7.1., Statsoft, Tulsa, OK, USA). Correlation was defined as moderate if the correlation coefficient r was less than 0.5, large if it was between 0.5 and 0.7, very large if it was between 0.7 and 0.9, and almost perfect above 0.9 (32, 33). Only correlations with coefficient r > 0.7, i.e., very large and almost perfect, were retained for the analysis. The slowest and fastest serves of the sample and of two players (one male and one female) were compared according to correlated parameters only for the acceleration phase, since it is during this phase that the racket acquires most of its velocity (21, 24).

## 3 Results

**Figure 1:**
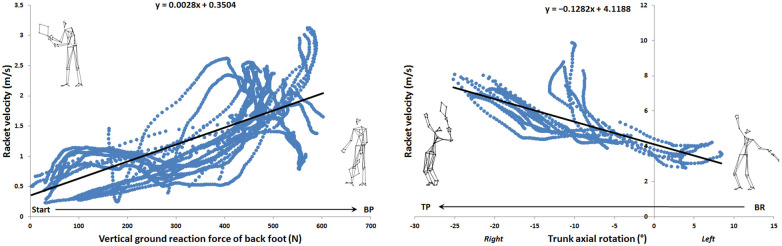
Linear correlation during release phase (left panel) and loading phase (right panel).

**Figure 2:**
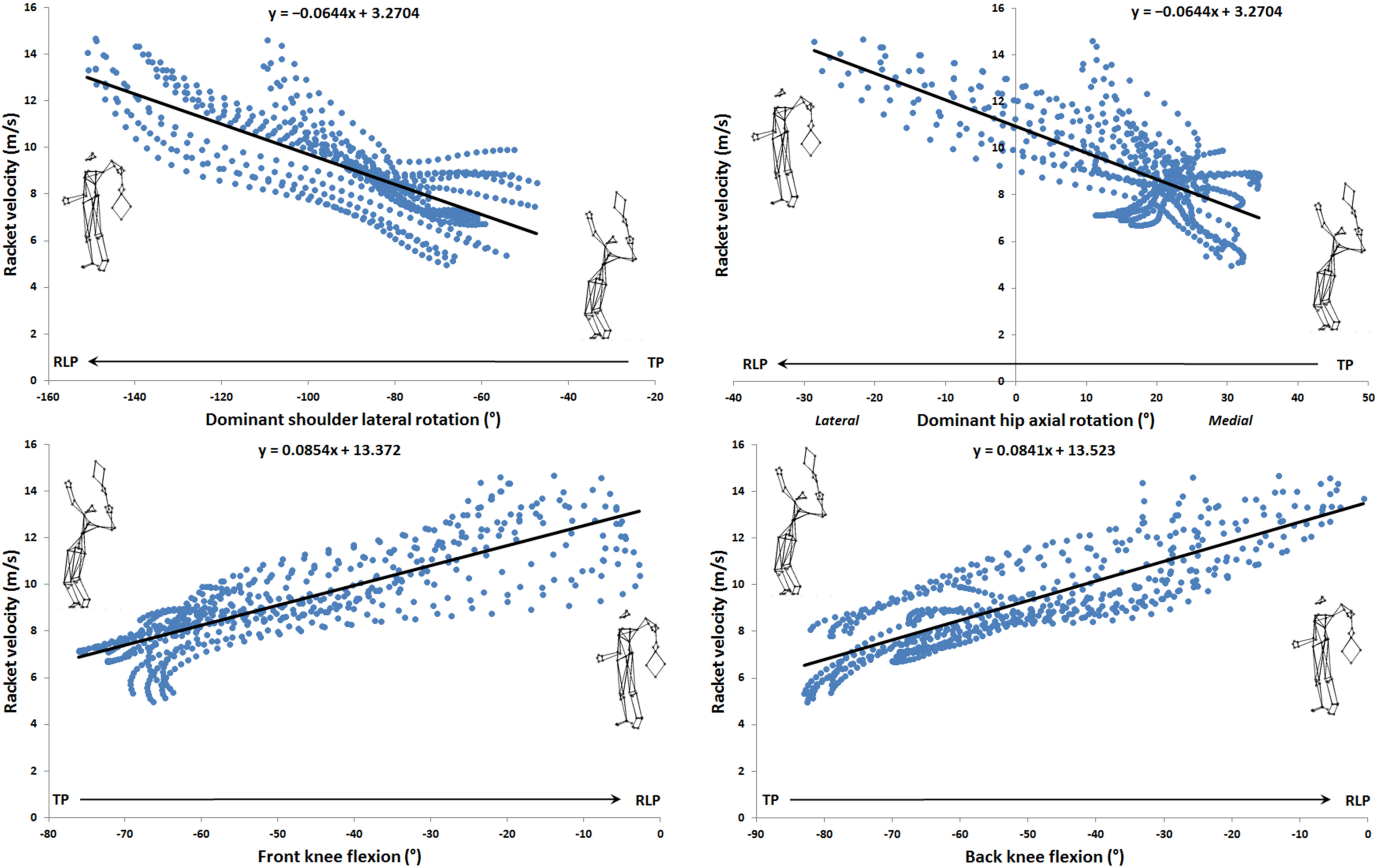
Linear correlation during cocking phase.

**Figure 3:**
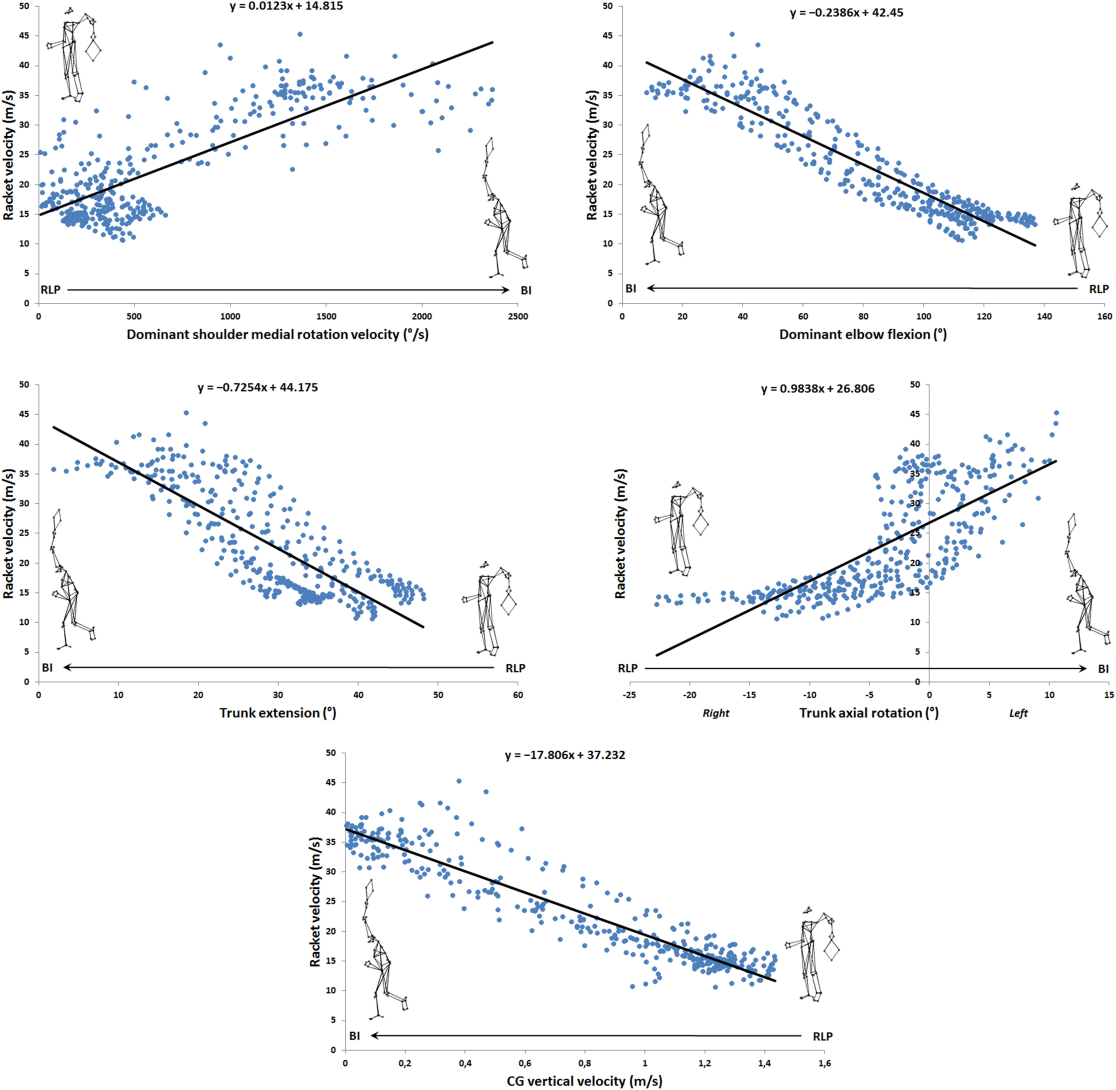
Linear correlation during acceleration phase.

Eleven significant correlations (r ≥ 0.7) were found: one in release backward phase (Figure 1, left panel), one during loading phase (Figure 1, right panel), four during cocking phase (Figure 2), and five during acceleration phase (Figure 3). Table 2 presents the values of the different correlation coefficients. Two negative almost perfect correlations were found in the acceleration phase between racket velocity and respectively dominant elbow flexion (r = −0.93) and CG vertical velocity (r = −0.94). Five joints (trunk, dominant shoulder, dominant elbow, dominant hip, and both knees) as well as CG vertical velocity and rear lower limb vertical reaction force correlated with racket velocity during the serve.

**Table 2:** Correlation coefficient between racket velocity and biomechanical parameters during the first four stages of tennis serve (4).

	Release				Loading		Cocking		Acceleration	
	Backward		Forward							
	r	*p*	r	*p*	r	*p*	r	*p*	r	*p*
CG vertical velocity	0.30	<0.001	−0.17	<0.001	0.08	0.029	0.69	<0.001	−0.94	<0.001
F vertical back lower limb	0.70	<0.001	−0.59	<0.001	0.55	<0.001	−0.40	<0.001	−0.05	0.309
Back knee flexion	0.08	<0.001	0.02	0.370	−0.18	<0.001	0.83	<0.001	−0.19	<0.001
Front knee flexion	0.30	<0.001	−0.64	<0.001	−0.56	<0.001	0.83	<0.001	0.01	0.789
Dominant shoulder mediolateral rotation	−0.13	<0.001	−0.62	<0.001	−0.64	<0.001	−0.77	<0.001	0.50	<0.001
Dominant shoulder mediolateral rotation velocity	0.39	<0.001	0.43	<0.001	−0.16	<0.001	0.44	<0.001	0.80	<0.001
Dominant elbow flexion	−0.28	<0.001	0.20	<0.001	0.58	<0.001	0.50	<0.001	−0.93	<0.001
Trunk flexion/extension	0.11	<0.001	0.52	<0.001	−0.44	<0.001	0.49	<0.001	−0.81	<0.001
Trunk axial rotation	−0.48	<0.001	0.10	<0.001	−0.71	<0.001	−0.26	<0.001	0.76	<0.001
Dominant hip mediolateral rotation	0.44	<0.001	0.28	<0.001	0.43	<0.001	−0.71	<0.001	−0.03	0.603

CG, center of gravity; F, Force; r, correlation coefficient.

Only very large (yellow) and almost perfect (green) correlation coefficients (r ≥ 0.7) are included in the analysis. Other coefficients are presented for information only.

**Figure 4:**
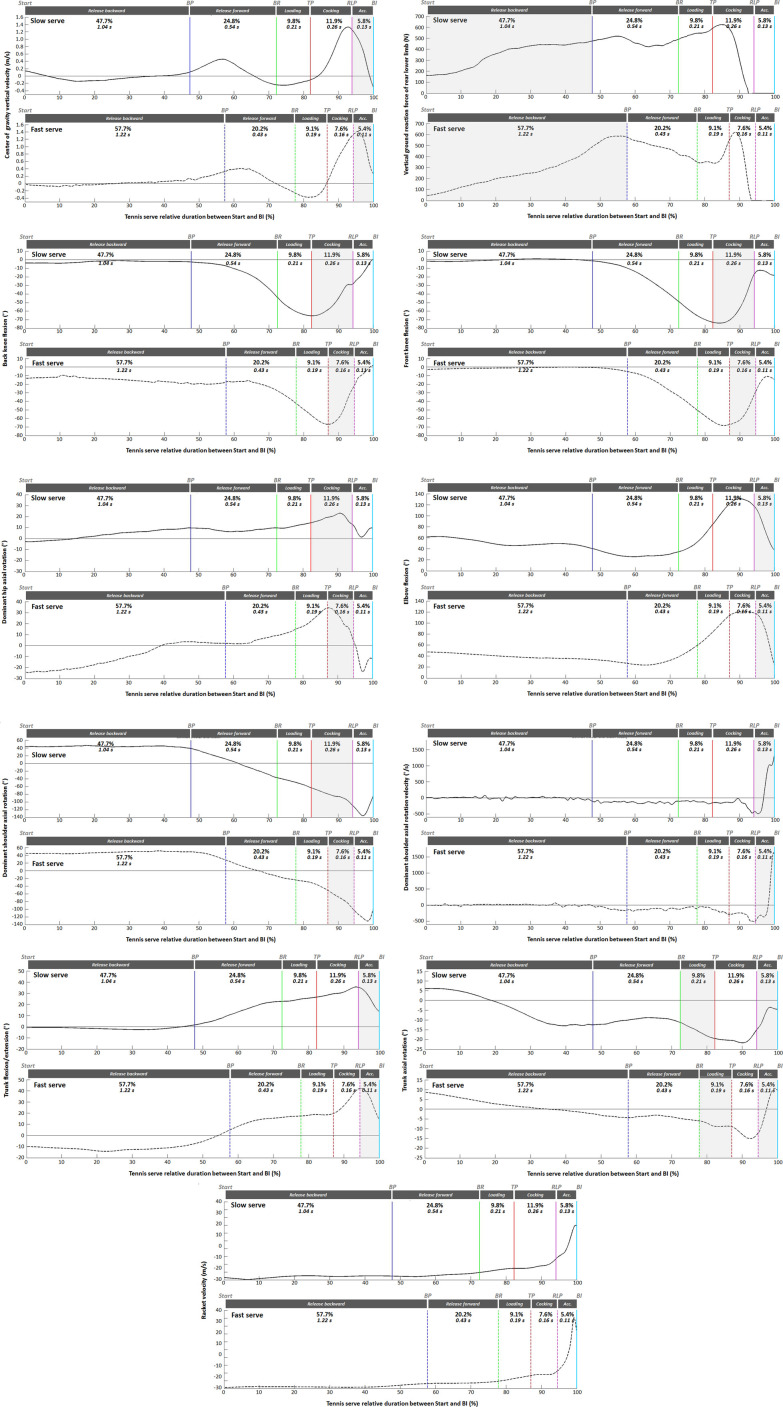
Temporal evolution of parameters correlated with racket velocity over the duration of a serve (between start and BI) for the slowest serve (top panel in solid line, subject 1, male) and the fastest serve (bottom panel in dotted line, subject 3, male) among 20 measured trials. The vertical lines represent the key points of interest. Shaded areas correspond to phases for which the parameter is correlated with racket velocity.

Figure 3 depicts the normalized time evolution of racket velocity and the 10 parameters correlated with it for all phases of the slow and fast serves. The absolute (in s) and relative (%) durations of each phase have been reported on each graph. The final graph in Figure 4 shows the evolution of racket velocity throughout the slow and fast serves. The peak racket velocity recorded during the acceleration phase was 34.89 m.s-1 and 45.43 m.s-1 for the slow and fast serves respectively. A difference in duration was observed between the slowest (2.18s) and fastest (2.11s) serves over the whole sample. The release backward phase was longer for the fast serve in absolute and relative values (1.22s vs. 1.04 s and 57.7% vs. 47.7% respectively) than for the slow serve. The durations of the other phases were systematically shorter for the fast serve. In relative values, combined with the release forward phase, the release phase accounts for 75% of the duration of a serve (slow or fast). The loading phase represents around 10% of the service, with a lower relative (9.8% vs. 9.1%) and absolute (0.21 s vs. 0.19 s) duration for the fast serve. Similar results were observed for cocking (11.9% vs. 7.6% and 0.26 s vs. 0.16 s). The acceleration phase is the shortest phase, representing less than 6% of the total serve duration, with an absolute duration of less than 15 ms. Lower values for fast serve were also observed (5.8% vs. 5.4% and 0.13 s vs. 0.11 s). It is during this phase that racket velocity increases the most.

The time evolution of the center of gravity vertical velocity shows two successive peaks. The first occurred during the release forward phase. It corresponds to the preparation of the ball toss. The second CG vertical velocity peak is located in the cocking phase for the slow serve (1.3 m/s), whereas it is in the acceleration phase for the fast serve (1.4 m/s).

The vertical force of the back foot increases during the release phase, with a relative maximum at the BR. The peak value was recorded during the cocking phase, corresponding to the jump impulse. The values were very close for both slow and fast serves (623.2 N vs. 619.0 N respectively).

Maximum flexion of both knees was observed at RLP for both serves, with close values (back knee flexion: −65.5° vs. −66.9; front knee flexion: −74.0° vs. −68.4° for slow and fast serve respectively). This is followed by an extension phase corresponding to the jump, ending with values close to 0° for the back knee and around −15° for the front knee.

For shoulder rotation, the serve starts with a medial rotation of 43.4° and 44.8° for slow and fast serves, which was maintained until BR. Medial rotation then decreases and becomes lateral rotation, with a peak reached during the cocking phase (between RLP and BI, −136.0° vs. −130.9° for slow and fast service respectively). Lateral rotation then decreases until BI (−84.8° vs. −100.7°). Shoulder velocity is close to 0°/s until BR, and then reflects the lateral rotation of the shoulder that occurs until after RLP. It becomes zero at the peak of maximum lateral shoulder rotation, and then increases rapidly until BI. This results in very quick medial rotation to hit the ball. Maximum values were recorded at BI with a difference between the two serves: 1,311.0°/s for the slow serve vs. 1,604.0°/s for the fast serve. For the elbow, flexion decreases throughout the release backward phase, then increases progressively during the release forward and loading phases, reaching a maximum value in the middle of the cocking phase (130.8° vs. 121.2° for slow and fast service respectively). Flexion then decreases quickly during the acceleration phase to BI (37.6° vs. 24.2°).

For the trunk, players were close to the neutral position or in slight flexion during the release backward phase, then progressively increase trunk extension until RLP (slow serve: 35.6° vs. fast serve: 42.0°). Extension decreases during the acceleration phase, reaching values of 13.6° and 14.2° respectively. For trunk rotation, from a slightly contralateral starting position, rotation progressively increases on the homolateral side (racket side) to reach a peak value during the cocking phase (between TP and RLP) of −21.7° for the slow serve and −15.1° for the fast serve. Homolateral rotation decreases during the acceleration phase. A notable difference appeared between the two serves at BI. For the slow serve, rotation was homolateral (−4.5°) whereas it was contralateral for the fast serve (9.6°).

The profiles of mediolateral hip rotations were similar for both serves. However, the ranges were different. For the slow serve, the initial position and at BI was close to the neutral position, whereas for the fast serve, rotation was lateral for these two key points. The peak value is observed at RLP for the fast serve (34.3°) and in the middle of the cocking phase (23.1°) for the slow serve.

Table 3 presents the values of the 5 parameters correlated with racket velocity during the acceleration phase for the slowest and fastest serves. In the fast service, the player hit the ball during the upward phase of the jump (positive CG vertical velocity), whereas it decreased in the slow service. Maximum medial shoulder rotation velocity was higher for the fast serve (1,604.0 vs. 1,311.0°/s) and elbow flexion lower at impact (24.2° vs. 37.6°). While trunk flexion/extension appeared identical for both serves, a notable difference was observed for axial rotation. Indeed, for the slowest serve, the player kept a homolateral rotation (that of RLP), whereas for the fastest serve the trunk rotation became contralateral. This resulted in a larger range of motion of trunk rotation for the fast serve (21.5° vs. 10.2°).

**Table 3:** Comparison of parameters correlated with racket velocity during the acceleration phase between a slow and a fast serve.

	Slow serve					Fast serve				
	RLP	BI	Range	Min	Max	RLP	BI	Range	Min	Max
CG vertical velocity (m/s)	1.2	−0.3	1.5	−0.3	1.2	1.4	0.3	1.1	0.3	1.4
Dominant shoulder mediolateral rotation velocity (°/s)	−435.2	1,311.0	1,746.2	−496.9	1,311.0	−492.3	1,578.5	2,070.8	−492.3	1,604.0
Dominant elbow flexion (°)	116.6	37.6	79.0	37.6	116.6	116.8	24.2	92.6	24.2	116.8
Trunk flexion/extension (°)	35.2	13.6	21.6	13.6	35.2	42.0	14.2	27.8	14.2	42.0
Trunk axial rotation (°)	−14.8	−4.5	10.2	−14.8	−3.5	−12.0	9.6	21.5	−12.0	10.6

CG, center of gravity; RLP, racket low point; BI, ball impact.

**Figure 5:**
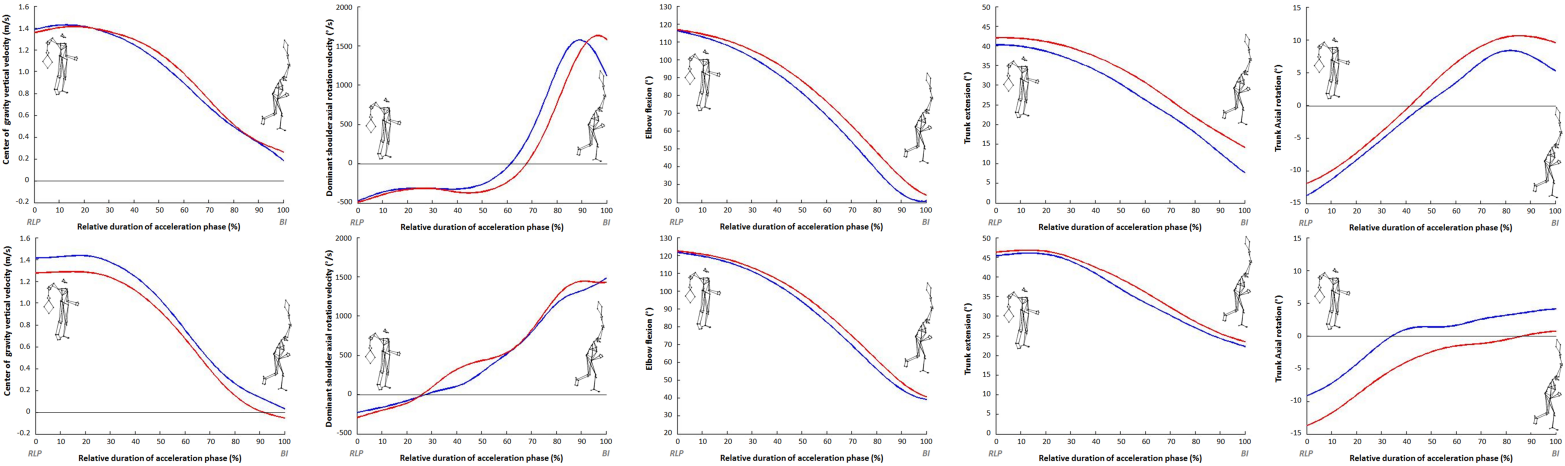
Example of relative temporal evolution of parameters correlated with racket velocity during acceleration phase (between RLP and BI) for the slowest and the fastest serve for the player 3 (male, top panels) and the player 2 (female, bottom panel).

Figure 5 displays the normalized time evolution of the acceleration phase for the 5 kinematic parameters very largely and almost perfectly correlated with racket velocity for the slowest and the fastest serve in 2 players, one male (top panels) and one female (bottom panels). Peak velocities for the slow serve were 132.4 km/h and 124.8 km/h and for the fast serve 163.5 km/h and 129.4 km/h respectively for both players.

Table 4 shows the values of 5 kinematic parameters (vertical velocity of the center of gravity, dominant shoulder mediolateral rotation velocity, dominant elbow flexion, trunk flexion/extension and trunk axial rotation) correlated very largely or almost perfectly with racket velocity at BI for the slowest and fastest serves of two players (player 3), one male and one female (player 2).

**Table 4:** Joint angle values at BI for the slowest and fastest tennis serves for 2 players.

	Player 3 (male)			Player 2 (female)		
	Slow	Fast	Diff	Slow	Fast	Diff
CG vertical velocity (m/s)	0.2	0.3	0.1	−0.3	−0.1	0.2
Dominant shoulder mediolateral rotation velocity (°/s)	1,115.8	1,578.5	462.7	1,282.7	1,283.5	0.8
Dominant elbow flexion (°)	20.7	24.2	3.5	10.9	9.9	−1.0
Trunk flexion/extension (°)	7.7	14.2	6.5	8.6	4.8	−3.9
Trunk axial rotation (°)	5.2	9.6	4.3	3.4	−2.6	−6.0

Diff, difference between fast and slow serve.

For player 3, the values of all parameters were higher for fastest serve. A significant difference was observed for dominant shoulder medial rotation velocity, with a value 40% higher for fastest serve (1,115.8 vs. 1,578.5°/s). Angular parameters differed by between 3.5 and 6.5°. The vertical velocity of the center of gravity was almost identical for both serves. The parameter that explains most of the difference in service speed is therefore the dominant shoulder medial rotation velocity for this player.

For player 2, the differences were small. With these 5 parameters, it is difficult to explain the difference in racket velocity observed between the two serves. We therefore looked at parameters in the acceleration phase that were highly correlated with racket velocity (0.5 < r < 0.7). We identified trunk flexion velocity, trunk rotation velocity and elbow flexion acceleration with correlation coefficients of r = −0.67, r = 0.57, and r = 0.57 respectively. For both trunk-related parameters, trunk axial rotation velocity is higher for fast serve (difference of 29.9°/s) while flexion velocity was lower (difference of −12.8°/s). For the elbow, the acceleration of elbow extension is much greater for the fast serve (difference of 27,894.1°/s^2^). For this player, the variation in racket velocity could be explained by the variation in elbow extension acceleration.

## 4 Discussion

The aim of this study was to investigate the correlations between kinematic and kinetic parameters and racket velocity during the tennis serve. An optoelectronic system and two force platforms were used to achieve this objective. The correlations were used to explain the differences between a slow and a fast serve evaluated through racket velocity. The originality of this work consists in taking into account reaction forces, the position and velocity of the center of gravity and body kinematics throughout all phases of the serve. The majority of correlations were observed in the cocking (4 correlations) and acceleration (5 correlations) phases. Vertical velocity of the center of gravity and elbow flexion during the acceleration phase were the two parameters that correlated most strongly (| r |>0.94 and | r |>0.93 respectively) with racket velocity.

To our knowledge, no studies have linked racket velocity to center of gravity and support forces. Nevertheless, some studies have used reaction forces to evaluate tennis serve. Elliott and Wood (19) provided the temporal profile of vertical and horizontal forces to compare two stance styles, i.e., foot-up vs. foot back. Comparisons were made at three instants of the serve, which do not correspond to the key points, and without information concerning the phases. Girard et al. (34) used Pedar insoles to compare the first and second serves for both stance styles. Data were presented for the entire duration of the stance, i.e., from the first foot movement to take-off, without any distinction between the different phases of the serve. The center of gravity is a parameter commonly used in biomechanics to study, at a single point, the general behavior of a body or an individual in a given activity, through the evolution of its trajectory, velocity and acceleration. In particular, it has been used in gait analysis for rehabilitation (35), robotic control (36), occupational ergonomics (37), and sports (38).

From a general point of view, eleven correlations, 9 very large and 2 almost perfect, were found over the entire serve, decomposed into 5 phases. The vertical force of the back foot was correlated with the racket velocity during release backward phase, the axial rotation of the trunk was correlated during the loading phase, the both knees flexion as well as the shoulder and hip mediolateral rotation were correlated in the cocking phase. Finally, vertical velocity of the center of gravity, shoulder mediolateral rotation velocity, elbow flexion and trunk flexion/extension and axial rotation were correlated in the acceleration phase. To our knowledge, only the works of Jacquier-Bret and Gorce (24) and Tanabe and Ito (21) found relationships. Tanabe and Ito (21) only investigated correlation at ball impact between upper limb joint angular velocities and horizontal racket velocity. Jacquier-Bret and Gorce (24) highlighted correlations between six kinematic parameters and racket velocity for the cocking and acceleration phases. However, other studies had already established relationships between kinematic parameters and specific phases under different experimental conditions. These include studies by Fett et al. (1) (effect of serve side, ad vs. deuce), Whiteside et al. (8) and Reid et al. (9) (effect of age) during the cocking and acceleration phases. In their work, the authors related the following kinematic parameters: peak separation angle, trunk tilt, trunk extension, trunk twist, trunk twist velocity, upper torso position, counter upper torso rotation, shoulder external rotation, shoulder horizontal flexion, shoulder internal (medial) rotation velocity, elbow flexion, elbow extension velocity, wrist flexion, wrist flexion velocity, hip vertical velocity, knee flexion, and knee extension velocity. The early phases of serve have been few studied. Fett et al. (1) evaluated the initial position through the position of the trunk and feet in relation to the service line for both sides. To our knowledge, no kinematic data is available in the literature for the release phase. The results of the correlations in the present study enabled us to identify the parameters involved in racket velocity by phase. This information is a valuable aid for coaches in training and performance optimization.

The correlated parameters were then used to study a slow and a fast serve. The underlying hypothesis is that the drop in performance could be explained by a variation in one or more parameters correlated with racket velocity in the different phases of the service. The analysis focused on the acceleration phase, as it is directly linked to the significant increase in velocity. In this phase, only five parameters were largely (0.7 < r < 0.9) or almost perfectly correlated (r > 0.9) with racket velocity: vertical velocity of the center of gravity, elbow flexion, trunk flexion/extension, trunk axial rotation, and shoulder medial rotation velocity. Table 3 shows the differences between the two serves. Joint range of motion is greater for fast than for slow serves, particularly for the elbow and axial rotation of the trunk. This indicates the importance of body kinematics for transferring velocity between the different segments during serve. Trunk axial rotation must be transmitted to the racket, and the elbow must be close to minimum flexion at ball impact. Some other studies reported low elbow flexion at BI around 20° (1, 17, 39). It seems important to maintain a slight flexion, as this would have a mechanical advantage for medial arm rotation and racket velocity (39). With regard to the shoulder, the medial rotation velocity is close to the values reported by Tubez et al. (40) and Zappala et al. (12) with a postural shirt but are lower than those reported in other studies (1, 13), probably due to the fact that the players examined had a higher serve velocity than those considered in our study. Moreover, the results of the present study (r = 0.8) are consistent with those of Tanabe and Ito (21), who also found a good correlation (r = 0.689) between shoulder medial rotation velocity and BI racket velocity. The authors reported that this velocity accounts for more than 41% of horizontal racket velocity. As presented in Table 3, shoulder medial rotation velocity is higher for the fast serve (1,311.0 vs. 1,604.0°/s), which largely explains the difference in performance. As regards GC vertical velocity, the values between the slow and fast serves were similar.

This observation could be applied directly to player 3 (male, Table 4). Indeed, a significant difference was found between the slow and fast serves for the medial shoulder rotation velocity (1,115.8 vs. 1,578.5°/s). The other kinematic parameters showed minimal differences between the two serves. The good reproducibility of joint angle could be explained by the fact that this player is of international level. The variation observed in shoulder velocity could be the main cause of the reduction in racket velocity for the slow serve. For player 2 (female), all the parameters correlated with racket velocity showed almost no difference between the two serve velocities (Table 4). It was therefore assumed that a difference could exist on parameters other than those with a very large correlation (r > 0.7). Thus, for this player, parameters with a correlation coefficient between 0.5 and 0.7 (high correlation) were analyzed (Table 2). Of the three parameters concerned (trunk flexion velocity, trunk rotation velocity, and elbow flexion acceleration), elbow flexion acceleration and trunk axial rotation velocity showed higher values for the fast serve (+27,894.1°/s^2^ and +29.9°/s respectively). These parameters could therefore explain the difference in racket velocity observed for this player. These results show that additional parameters can be considered (r > 0.5, Table 2) in a player of good level for whom differences do not appear for strongly correlated parameters. Individualized analysis based on parameters correlated with racket velocity is the second objective of the present study. It highlighted the differences in amplitude of values between a slow and a fast serve. As a result, a coach can use these parameters to increase a player's serve speed. For example, for player 3, the aim should be to increase shoulder rotation speed to increase serve speed. So, in addition to technical work, exercises could include strengthening the shoulder's medial rotator muscles (i.e., teres major, anteroir deltoid, subscapularis, pectoralis major, latissimus dorsi).

In practical terms, this study identified 11 parameters correlated with racket velocity, distributed over the different phases of the tennis serve. The vertical reaction force of the rear foot characterizes the release backward phase, in which the player moves backwards. During the loading phase, a large trunk axial rotation on the dominant side seems to be preponderant for high racket velocity, suggesting a positioning adapted to the desired direction of the serve. During the cocking phase, the three joint angles to be privileged are knee flexion and lateral rotation of the shoulder and hip. Care should be taken to ensure knee flexion close to 75° (23) and significant shoulder lateral rotation [>140° (9, 12)]. During this phase, lateral rotation of the dominant hip should be performed between TP and RLP. Finally, for the acceleration phase, 5 parameters could be considered to optimize the racket velocity. First, vertical velocity of the center of gravity is negatively correlated with racket velocity, suggesting that it would be preferable to hit the ball when the center of gravity velocity is close to 0 m.s^−1^, i.e., at the maximum height of the jump. Similarly, the negative correlation between elbow flexion and trunk flexion suggests that it is important to achieve trunk flexion from an extended position, as well as elbow extension from the flexed position achieved at RLP. Both parameters should be close to neutral at impact to ensure high racket velocity (Figure 3). Some authors, however, recommend a slight elbow flexion at the moment of BI to prevent the impact of the anterior part of the ulnar olecranon in the olecranon fossa of the humerus, which reduces the risk of pathologies such as osteophytes, osteochondritis dissecans, or loose body formation (39, 41). Contralateral trunk rotation also contributes significantly to the generation of racket velocity. It seems necessary to hit the ball when the trunk has passed neutral and begun to rotate on the opposite side to the arm holding the racket without reaching maximum rotation. Finally, it is essential to aim for a high shoulder medial rotation velocity up to BI [about 2,000°/s measured in adults (8)] to maximize racket velocity. All these correlated kinematic parameters could be used by trainers to improve tennis serves.

A few limitations could be addressed. The study was carried out without considering the different types of serve (flat, slice, kick, top spin) or stance styles (foot-up vs. foot-back). Taking these parameters into account could enable us to refine the correlations and adapt them to different techniques of serve. On the other hand, the study only included young adults in the correlations. Extending the study to a larger cohort, taking age and level of expertise into account, would help generalize the results further. In the analysis, correlations were defined using a linear model to facilitate their exploitation. Quadratic models might have been more appropriate for some variables, and could have brought out new parameters.

## 5 Conclusion

A detailed analysis of whole-body kinematics and center of gravity coupled with ground reaction forces was carried out over all phases of the tennis serve, using an optoelectronic system and two force platforms. Eleven parameters were identified as being very largely and almost perfectly correlated with racket velocity: vertical ground reaction force of back foot in release backward, trunk axial rotation during loading phase, back and front knee flexions, dominant shoulder and hip mediolateral rotation during cocking phase, and CG vertical velocity, dominant shoulder medial rotation velocity, dominant elbow flexion, trunk flexion/extension and axial rotation during acceleration phase. Analysis of these parameters enables differentiation between a slow and a fast serve. All these correlated kinematic parameters constitute information that coaches, instructors and athletes can use to improve, optimize or teach the tennis serve.

## Data Availability

The raw data supporting the conclusions of this article will be made available by the authors, without undue reservation.
